# Metabolomics profiling reveals differential adaptation of major energy metabolism pathways associated with autophagy upon oxygen and glucose reduction

**DOI:** 10.1038/s41598-018-19421-y

**Published:** 2018-02-05

**Authors:** Katja Weckmann, Philip Diefenthäler, Marius W. Baeken, Kamran Yusifli, Christoph W. Turck, John M. Asara, Christian Behl, Parvana Hajieva

**Affiliations:** 1 Institute of Pathobiochemistry, Johannes Gutenberg University, Medical School, Duesbergweg 6, 55099 Mainz, Germany; 2 Department of Translational Research in Psychiatry, Max Planck Institute of Psychiatry, Kraepelinstr. 2–10, 80804 Munich, Germany; 3 Division of Signal Transduction/Mass Spectrometry Core, Beth Israel Deaconess Medical Center, Boston, Massachusetts, USA and Department of Medicine, Harvard Medical School, Boston, Massachusetts, USA

## Abstract

The ability of cells to rearrange their metabolism plays an important role in compensating the energy shortage and may provide cell survival. Our study focuses on identifing the important adaptational changes under the conditions of oxygen and glucose reduction. Employing mass spectrometry-based metabolomics in combination with biochemistry and microscopy techniques we identified metabolites, proteins and biomolecular pathways alterations in primary human IMR90 fibroblasts upon energy deficits. Multivariate statistical analyses revealed significant treatment-specific metabolite level and ratio alterations as well as major energy metabolism pathways like ‘glycolysis’, ‘pentose phosphate pathway’, ‘mitochondrial electron transport chain’ and ‘protein biosynthesis (amino acids)’ indicating an activation of catabolism and reduction of anabolism as important mechanisms of adaptation towards a bioenergetic demand. A treatment-specific induction of the autophagic and mitophagic degradation activity upon oxygen reduction, glucose reduction as well as oxygen-glucose reduction further supports our results. Therefore, we suggest that the observed alterations represent an adaptive response in order to compensate for the cells’ bioenergetics needs that ultimately provide cell survival.

## Introduction

A number of pathological conditions are accompanied by cellular bioenergetic perturbations mainly triggered by oxygen reduction (OR) and glucose reduction (GR). For instance, energy imbalances observed by unexplained weight loss is a frequently observed clinical finding for patients suffering from cancer, heart failure, Alzheimer’s and Parkinson’s disease^1^. However, the basic bioenergetic adaptation mechanism remains elusive. Adaptation to glucose and oxygen reduction requires biochemical and genetic responses culminating in an acute and/or chronic metabolic rearrangement in order to meet the cells’ bioenergetic needs. Multiple pathways may alter under nutrient stress, as glucose and oxygen are the major regulators of the cellular core metabolism. Eukaryotic cells seem to generally adapt to nutrient deprivation by modifying glycolytic and mitochondrial metabolism that are important entities of the cellular core metabolism^2^. However, the decisive adaptive pathways ensuring cell survival remain elusive.

Oxygen and glucose are essential for the mitochondrial energy metabolism in order to generate energy in form of adenosine triphosphate (ATP) through the oxidative phosphorylation (OXPHOS). Ischemia, tissue damage and poorly vascularized cancer can restrict oxygen supply leading to metabolic stress and potentially apoptosis^3^. AMP activated protein kinase (AMPK) is the major energy status sensor that promotes and inhibits catabolic and anabolic pathways, respectively, to generate ATP. High AMP and ADP levels activate the AMPK, whereas increased levels of ATP inhibit the enzyme^4–8^. The mammalian target of rapamycin (mTOR) is a central regulatory nexus that integrates physiological signals from different cellular signaling cascades, thereby modulating anabolic versus catabolic pathways in response to nutrients, growth factors, and the cellular energy status. mTOR controls several metabolic pathways at the transcriptional, translational, and posttranslational level including ‘glycolysis’, the ‘pentose phosphate pathway’, and OXPHOS by positively modulating mitochondrial biogenesis and activity^9–12^.

Enhanced autophagic and mitophagic activity buffer metabolic stress most likely by degrading intracellular energy reserves like lipids, glycogen, and proteins to generate amino acids, nucleotides, fatty acids, and sugars that are recycled into metabolic pathways^13^. Thus, autophagy and mitophagy also contribute in maintaining the cellular energy balance. Another bioenergetic adaptive response is mostly described for cancer cells in hypoxic microenvironments: Warburg firstly proposed a metabolic reprogramming from oxidative phosphorylation (OXPHOS) to aerobic glycolysis in order to produce ATP and metabolic intermediates^14^. Therefore, changes in glucose and oxygen availability induce an adaptive response that may allow for extended survival.

Metabolomics profiling analysis is an important tool to generate a cellular metabolic fingerprint under stress conditions. Alterations provide significant and novel knowledge towards the cells’ metabolism adjustment to counter-balance the energy defect^15–18^.

In our present study, we aim to analyze the crosstalk between the energy homeostasis with mitophagy and autophagy. We employed primary human IMR90 fibroblasts to investigate the effect of OR, GR and oxygen-glucose reduction (OGR) by metabolomics profiling analyses, biochemical and microscopic analyses. Our goal was to identify significantly altered metabolite levels and ratios, enriched biochemical pathways and biosignatures responsible for the restoration of the cellular energy demand providing cell survival.

Our study demonstrates that cells deprived in GR, OR and OGR demonstrate a treatment-specific pattern of metabolic changes, which manifests by altering major energy metabolism pathways. Moreover, our metabolomics profiling approach reveals a differential treatment-induced adaptation mechanism associated with autophagy and mitophagy.

## Results

### Cell survival analysis

Glucose and oxygen are major energy sources and regulators of metabolism. Therefore, a long-term withdrawal of glucose and oxygen may have an impact on cell morphology and survival in cell culture conditions. Microscopic examination of cells after 24 h of OR, GR and OGR revealed no significant changes in morphology. Western blotting analysis of the cleaved Caspase 3, a well-established marker of apoptosis, revealed no signs of apoptosis upon OR, GR and OGR (Fig. S1).

### Metabolomics profiling analyses

Altogether 292, 287 and 292 metabolites were quantified in primary human IMR90 fibroblasts comparing OR, GR and OGR with control, respectively (Table S1). We combined three different standard multivariate statistical methods to achieve robust metabolomics results. Generally, statistical tests including principal component analyses (PCA), partial least squares-discriminant analysis (PLS-DA) and significance analysis of microarrays and other -omics datasets (SAM) are employed in order to analyze metabolite profiles. PCA and PLS-DA are statistical tests in order to analyze treatment patterns and group separation. Additionally, the PLS-DA variable influence of projection (VIP)-score ranks metabolites according to their importance for group separation. SAM can be employed to analyze statistical significant metabolites comparing different treatment groups.

**Figure 1:**
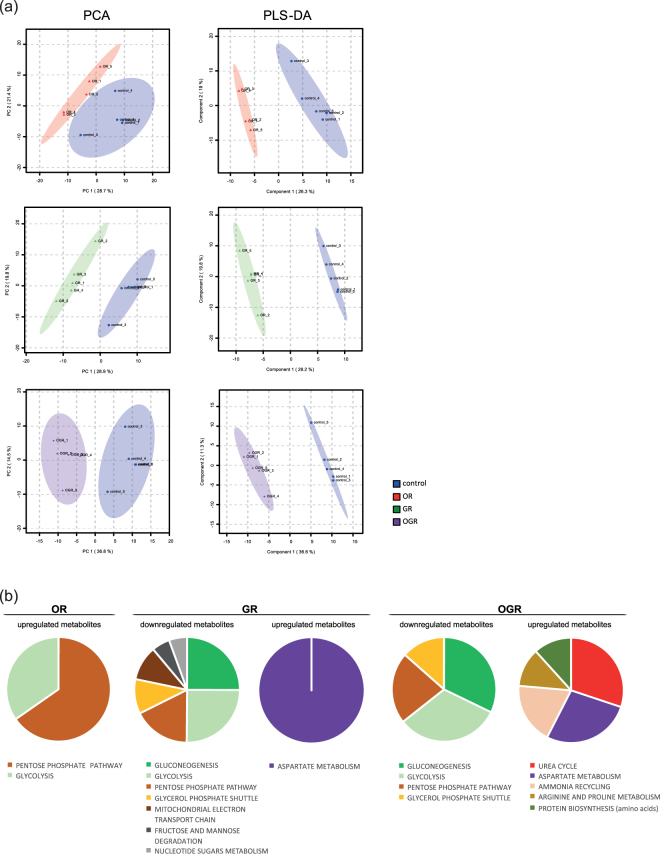
(**a**) Multivariate unsupervised principal component analysis (PCA) and supervised partial least squares-discriminant analysis (PLS-DA) comparing control (21% oxygen and 4.5 g/L glucose) with oxygen reduction (OR, 1% oxygen and 4.5 g/L glucose), glucose reduction (GR, 21% oxygen and 0 g/L glucose) and oxygen-glucose reduction (OGR, 1% oxygen and 0 g/L glucose) using all quantified metabolites for each treatment group. All R^2^, Q^2^ and accuracy values indicate good and well fit PLS-DA models (OR: R^2^ = 0.997, Q^2^ = 0.681, accuracy = 0.90; GR: R^2^ = 0.999, Q^2^ = 0.867, accuracy = 1.0; OGR: R^2^ = 0.999, Q^2^ = 0.873, accuracy = 1.0). N = 5 per group. (**b**) Pie chart representation of the Metabolite set enrichment analysis (MSEA) under OR, GR and OGR. The size of the pie chart is represented by –log10FDR (adjusted p-value) obtained by MSEA. N = 5 per group.

In the present study, metabolite profiles separated control from OR, GR and OGR in the PCA and PLS-DA (Fig. 1). We further assessed the quality of the data by calculating Q^2^ values indicating good and predictive PLS-DA models for OR, GR and OGR (OR: Q^2^ = 0.68, R^2^ = 1.00, Accuracy = 0.90; GR: Q^2^ = 0.87, R^2^ = 1.00, Accuracy = 1.00; OGR: Q^2^ = 0.87, R^2^ = 1.00, Accuracy = 1.00).

Additionally, 45, 47 and 89 metabolites were significantly altered in OR, GR and OGR, respectively. For OR 19 and 26, GR 15 and 32, and OGR 45 and 44 metabolites were up- and downregulated, respectively (Tables S2–S4). Furthermore, we analyzed overlapping metabolites for all conditions. Therefore, we compared significantly up- and downregulated metabolites of each treatment with a VENN-diagram. Interestingly, there was no overlap for OR and GR observed for the significantly up-regulated metabolites. Decreased metabolites were overlapping for all conditions. However, OR seems to be largely differing from GR: Although OR shares 7 and 13 metabolites with GR and OGR, respectively, GR shares 27 metabolites with OGR (Fig. S2).

Next, the statistically significant altered up- and downregulated metabolites from OR, GR and OGR were interrogated for overrepresented (enriched) biomolecular pathways.

OR-upregulated metabolites statistically significant enriched the ‘pentose phosphate pathway’ and ‘glycolysis’, whereas OR-downregulated metabolites did not statistically enrich any pathways. Furthermore, the ‘aspartate metabolism’ was enriched and upregulated upon GR, whereas ‘gluconeogenesis’, ‘glycolysis’, ‘pentose phosphate pathway’, ‘glycerol phosphate shuttle’, ‘mitochondrial electron transport chain’, ‘fructose and mannose degradation’ and ‘nucleotide sugars metabolism’ were significantly overrepresented and decreased. OGR showed a significant enrichment and downregulation of the ‘gluconeogenesis’, ‘glycolysis’, ‘pentose phosphate pathway’ and ‘glycerol phosphate shuttle’, whereas the ‘urea cycle’, ‘aspartate metabolism’, ‘ammonia recycling’, ‘arginine and proline metabolism’ and ‘protein synthesis (amino acids)’ were significantly overrepresented and elevated (Fig. 1 and Fig. S6).

Interestingly, ‘glycolysis’, ‘pentose phosphate pathway’, ‘gluconeogenesis’, ‘glycerol phosphate shuttle’ and ‘aspartate metabolism’, overlap in all conditions. Comparing OR with GR and OGR, we uncovered an opposite regulation of the ‘glycolysis’ and ‘pentose phosphate pathway’ (Fig. 1 and Fig. S6).

### Analysis of major energy metabolism pathways and the energy status upon OR, GR and OGR

The ‘glycolysis’ and ‘citric acid cycle’ consist of a series of biochemical reactions to generate high levels of energy in form of ATP through the connected OXPHOS. The succinate dehydrogenase subunit A, the enzyme metabolizing succinic acid to fumaric acid connects the ‘citric acid cycle’ to the ‘OXPHOS’. This process is also known as mitochondrial respiration and consists of 5 complexes. These are integrated into the mitochondrial inner membrane and transport electrons from NADH + H^+^ and succinic acid from the complexes I and II, respectively. The electron transport results from pumping H^+^ from the mitochondrial matrix into the intermembrane space of the mitochondria resulting in a mitochondrial membrane potential creating an electrochemical gradient. The complex V then uses this energy in order to produce ATP from adenosine diphosphate (ADP) by releasing H^+^ back into the mitochondrial matrix.

**Figure 2:**
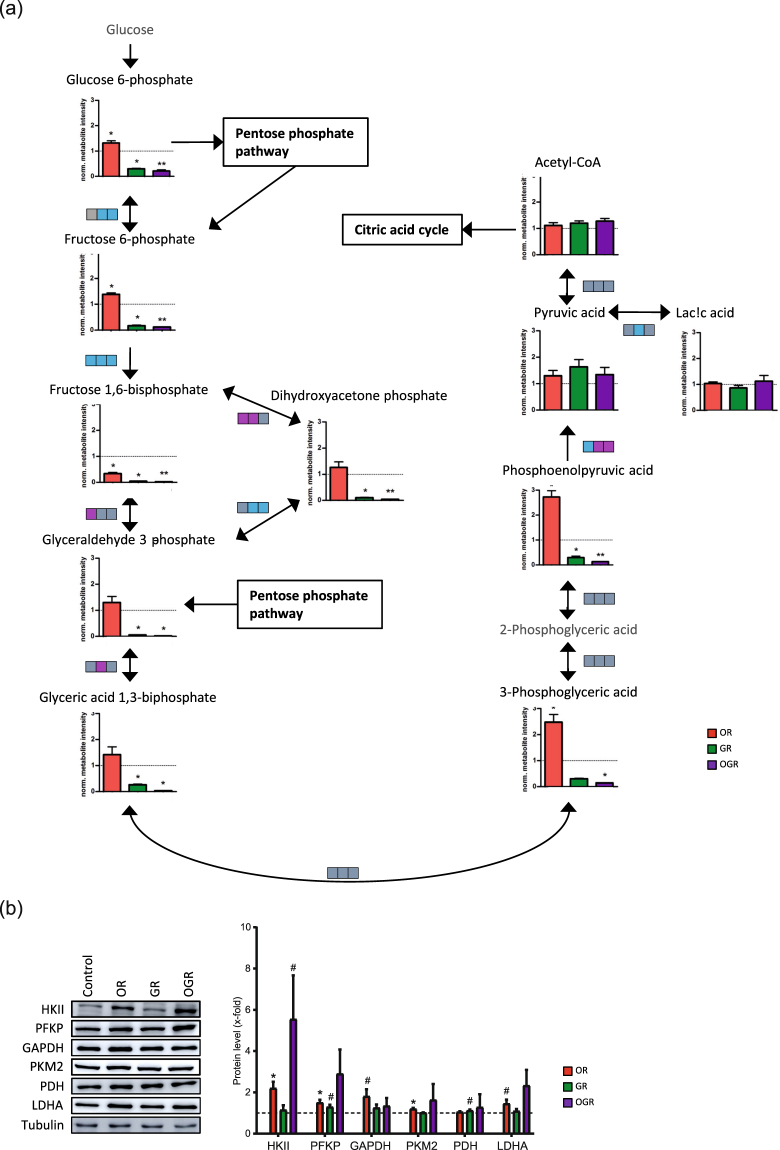
(**a**) Glycolysis metabolite level and metabolite ratio analyses upon OR, GR and OGR. Metabolite ratios are indicated by boxes and each box represents a different treatment group (from left to right OR, GR and OGR). Significant metabolite ratio differences are illustrated in pink (increased ratio), blue (decreased ratio) and gray (unchanged ratio). Significant metabolite ratios were calculated by Student’s t-test (compare Fig. S3). Significant metabolite levels were calculated by SAM: *FDR ≤ 0.05; **FDR ≤ 0.01; ***FDR ≤ 0.001. N = 5 per group. (**b**) Western Blotting analyses of glycolytic enzymes. IMR90 cells were subjected to OR, GR and OGR for 24 h. After that cells were harvested and total cell lysate was analyzed using Western blotting and immunodetected with indicated antibodies. Tubulin was used as a loading control. For the densitometric quantification of the immunoreactive bands the absolute values measured were first normalized to tubulin and the resulting values to the control, which was set as 1. # ≤ 0.10, *p ≤ 0.05; **p ≤ 0.01, ***p ≤ 0.001. P-values were determined by one-way analysis of variance (ANOVA) with post-hoc Tukey honestly significant difference (HSD) test. Error bars represent s.e.m. N = 3 per group.

**Figure 3:**
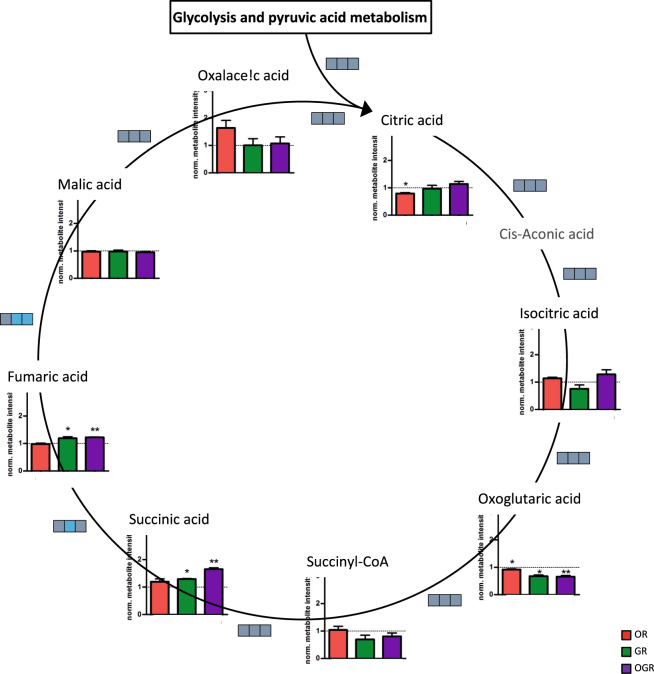
Citric acid cycle metabolite level and metabolite ratio analyses upon OR, GR and OGR. Metabolite ratios are indicated by boxes and each box represents a different treatment group (from left to right OR, GR and OGR). Significant metabolite ratio differences are illustrated in pink (increased ratio), blue (decreased ratio) and gray (unchanged ratio). Significant metabolite ratios were calculated by Student’s t-test (compare Fig. S4). Significant metabolite levels were calculated by SAM: *FDR ≤ 0.05; **FDR ≤ 0.01; ***FDR ≤ 0.001. N = 5 per group.

Glycolytic metabolites such as glucose 6-phosphate and fructose 6-phosphate levels were significantly elevated in OR and decreased in GR and OGR. Fructose 1,6-phosphate levels were downregulated in all conditions whereas dihydroxyacetone-phosphate, glyceraldehyde 3-phosphate and glyceric acid 1,3-bisphoaphate showed no alteration in GR, but decreased levels in OR and OGR. 3-Phosphoglyceric acid was significantly increased and decreased during GR and OGR, respectively. Phosphoenolpyruvic acid was also significantly elevated in OR, but downregulated in GR and OGR. Interestingly, pyruvic acid, acetyl-CoA and lactic acid metabolite levels showed no significant alterations (Fig. 2a). Acetyl-CoA is then transferred into the mitochondria and introduced into the ‘citric acid cycle’. Citric acid metabolite levels were significantly lowered upon OR. Oxoglutaric acid was significantly downregulated in all three conditions. GR and OGR significantly increases succinic acid as well as fumaric acid (Fig. 3).

**Figure 4:**
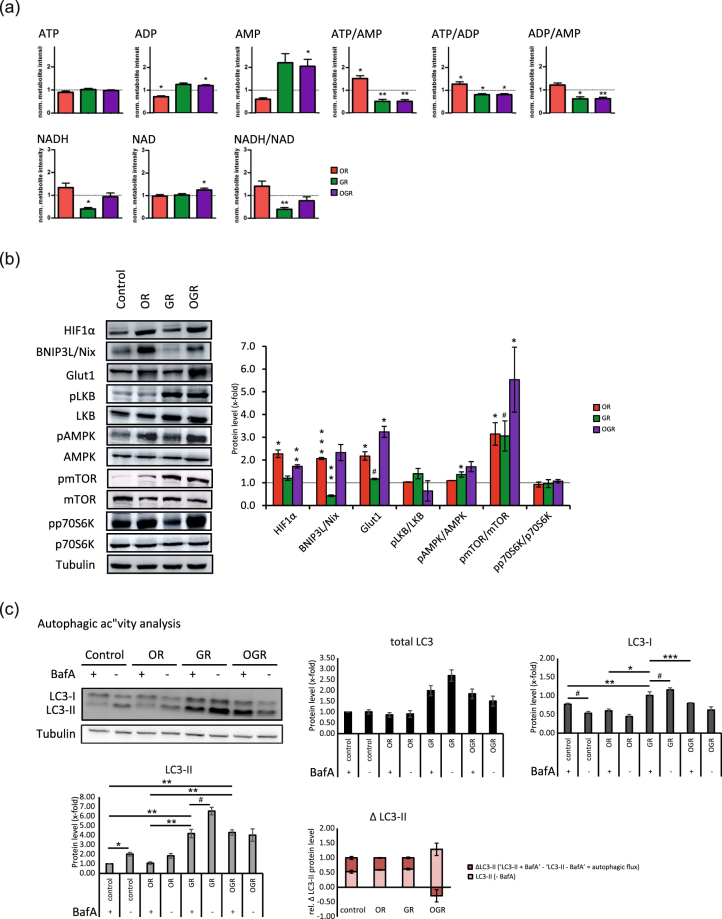
(**a**) Energy-status analyses of ATP, ADP, AMP, NAD and NADH with the related metabolite ratios ATP/AMP, ATP/ADP, ADP/AMP, and NADH/NAD. *p ≤ 0.05; **p ≤ 0.01. P-values were determined by Student’s t-test. Error bars represent s.e.m. N = 5 per group. (**b**) Western Blotting analyses of markers for GR and OR and proteins involved in the cellular energy metabolism. IMR90 cells were subjected to OR, GR and OGR for 24 h. After that cells were harvested and total cell lysate was analyzed using Western blotting and immunodetected with indicated antibodies. Tubulin was used as a loading control. For the densitometric quantification of the immunoreactive bands the absolute values measured were first normalized to tubulin and the resulting values to the control, which was set as 1. # ≤ 0.10, *p ≤ 0.05; **p ≤ 0.01, ***p ≤ 0.001. P-values were determined by one-way analysis of variance (ANOVA) with post-hoc Tukey honestly significant difference (HSD) test. Error bars represent s.e.m. N = 3 per group. (**c**) Autophagic degradation activity analyses upon OR, GR and OGR compared to control measured by Western blotting analyzing LC3 and LC3-II protein levels and LC3-II protein turnover with and without BafA. The autophagic degradation activity (autophagic flux) was determined by the following calculation: ΔLC3-II = ‘LC3-II + BafA’ - ‘LC3-II - BafA’. Tubulin was used as a loading control. # ≤ 0.10, *p ≤ 0.05, **p ≤ 0.01. P-values were determined by one-way ANOVA with post-hoc Tukey HSD test. Error bars represent s.e.m. N = 3 per group.

Metabolite ratios can indicate alterations in enzyme activity or expression rate. ‘Glycolysis’ and ‘citric acid cycle’ metabolite ratios (Figs 2a, 3 and Fig. S3) are indicated by boxes. Each box represents a single condition (from left to right: OR, GR and OGR). Metabolite ratio differences are illustrated in pink (increased) and blue (decreased). GR and OGR reduces the fructose-6-phosphate/glucose-6-phosphate ratio. The fructose-1,6-phosphate/fructose-6-phosphate metabolite ratio showed a significant downregulation in all conditions. The fructose 1,6-bisphosphate/dihydroxyacetone phosphate metabolite ratio was upregulated upon OR and GR, whereas glyceraldehyde 3-phosphate/dihydroxy-acetone-phosphate ratios were downregulated in GR and OGR. The glyceraldehyde 3-phosphate/fructose 1,6-bisphosphate ratio was significantly increased upon OR. The pyruvic acid/phosphoenolpyruvic acid ratio was significantly lower upon OR and increased in GR and OGR. GR significantly decreases the lactic acid/pyruvic acid metabolite ratio (Fig. 2a and Fig. S3). GR significantly decreased citric acid cycle fumaric acid/succinic acid metabolite ratio and GR and OGR significantly lowered the malic acid/fumaric acid (Fig. 3 and Fig. S4). In addition, we validated metabolite ratio alterations by investigating related glycolytic protein levels. OR increased hexokinase II (HKII), phosphofructokinase (PFKP), pyruvate kinase isoform M2 (PKM2) and tended to elevate glyceraldehyde 3-phosphate dehydrogenase (GAPDH) and lactate dehydrogenase (LDHA) protein levels. Additionally, OGR and GR tended to increase HKII and pyruvate dehydrogenase (PDH) protein levels, respectively. However, the effect size of PKM2 and GAPDH upon OR and PFKP upon GR are small (Fig. 2b). Our observations are not completely in line with the calculated metabolite ratio differences. However, for instance, the enzymatic activity of GAPDH is regulated by a decreased NADH/NAD ratio^19^. Indeed, we observed a significant decrease of the NADH/NAD ratio under GR; however, not upon OR and OGR (Fig. 4a and Fig. S5). Therefore, we conclude that our observed metabolite ratio changes not necessarily lead to protein level changes, but can rather indicate enzymatic activity alterations.

We further analyzed the energy status upon OR, GR and OGR (Fig. 4a). The ‘citric acid cycle’ and ‘glycolysis’ are affected upon GR, OR and OGR and these pathways ultimately generate ATP predominantly through the connected ‘OXPHOS’. ATP metabolite levels were unchanged during all conditions. GR and OGR significantly reduce and increase the ADP levels, respectively. OGR significantly increases the AMP levels. The ATP/AMP and ATP/ADP metabolite ratios were significantly increased in OR and downregulated in GR and OGR (Fig. 4a).

In addition to our metabolomics analyses, we also assessed protein levels of the hypoxia inducible factor 1 α (HIF1α), an established marker for OR and implicated in cancer. Upon OR, HIF1α leads to the expression of several target genes including glucose transporter 1 (Glut1), glycolytic enzymes and the mitophagy marker BNIP3L/Nix^20–27^. As expected, HIF1α protein levels were significantly elevated under OR and OGR. Bnip3L/Nix was significantly increased and reduced upon OR and GR, respectively. Glut1 protein levels were significantly increased under OR and OGR and tended to be elevated upon GR (Fig. 4b). As expected and described above many glycolytic enzymes were elevated upon OR and OGR (Fig. 2b).

Maintenance of intracellular energy status is essential for cellular viability and function. Therefore, activation of pro-survival energy sensors might exert partially through the increase of energy sensor proteins. To test this hypothesis, we examined major biomolecular energy sensor protein levels of liver kinase B1 (LKB1) and AMP activated protein kinase (AMPK)^28^. The phosphorylated forms of LKB1 (pLKB1) was increased under GR and OGR and pAMPK upon all three conditions. However, the unphosphorylated forms of LKB and AMPK are also elevated thus only significantly altering the pAMPK/AMPK ratio upon GR (Fig. 4b). mTOR is a an important regulator integrating external and internal signals, such as growth factors, amino acids, glucose and energy status to maintain growth and metabolism^29,30^. mTOR is primarily involved in protein synthesis, cellular metabolism and further inhibits autophagy. We analyzed mTOR as well as its direct target ribosomal protein S6 kinase beta-1 (p70S6K) by assessing protein levels of the active phosphorylated forms of mTOR (pmTOR) and p70S6K (pp70S6K). Interestingly, the pmTOR/mTOR ratio significantly increased upon OR, OGR and tended to be elevated upon GR, whereas the pp70S6K/p70S6K protein levels are unchanged (Fig. 4b).

### Treatment-induced alterations of the autophagic and mitophagic degradation activity

AMPK is a well-known activator and mTOR a suppressor of autophagy, which is accompanied by an increase and decrease of the autophagic degradation activity (autophagic flux), respectively^31^. pAMPK phosphorylates GAPDH subsequently leading to SIRT1 activation and therefore an induction of autophagy^19^. Autophagy is a protective degradative and catabolic process removing misfolded, unfolded or heavily oxidized proteins and damaged organelles that can disrupt cellular homeostasis^32^. Cellular survival under nutrient shortage is supported by recycling and providing bioenergetics and biosynthetic substrates^13^. Indeed, we measured a high accumulation of amino acids under OGR (Fig. 1b and Fig. S6B).

Autophagy plays an important role in accelerated degradation of mitochondria (mitophagy) in *in vivo* models of hypoxia^33^. Accordingly, proteins and mitochondria sequester into double-membrane vesicles, called autophagosomes, which subsequently fuse with lysosomes, forming autophagolysosomes. Subsequently, the breakdown products generated by hydrolytic enzymes in the lysosome are recycled for macromolecular intermediate synthesis and ATP generation. LC3-I is processed to its phosphatidylethanolamine-conjugated form LC3-II and then recruited to autophagosome membranes, which typically correlates with the number of autophagosomes^34,35^. Due to a high LC3-II protein turnover rate, we used the lysosomal vacuolar H + ATPase (v-ATPase) inhibitor Bafilomycin (BafA). Therefore, autophagy is blocked by inhibiting fusion of autophagosomes with lysosomes^36^. A subsequent LC3-II accumulation is observed due to an increased autophagic degradation activity or autophagosome formation^37,38^. The autophagic degradation activity can be determined by detecting the LC3-II protein turnover.

The above results prompted us to investigate whether OR, GR and OGR exert any effects on the autophagic and mitophagic activity by detecting LC3-II and BNIP3L/Nix, respectively. Interestingly, GR already increases LC3-I protein levels without BafA treatment. Upon BafA treatment LC3-I protein levels decrease and elevate comparing the control and GR, respectively. GR and OGR without BafA treatment increases LC3-II protein levels and further tended to elevate upon GR with BafA (Fig. 4c). However, OGR does not lead to new autophagosomes as BafA does not lead to an elevation of LC3-II protein levels. Additionally, the relative ΔLC3-II protein levels showed no significant alterations upon OR, GR and OGR (Fig. 4c). Taken together, GR leads to an induction of autophagy by an increasing the formation of autophagosomes, whereas OGR might rather lead to an impaired autophagic degradation as the autophagosome number does not increase with the addition of BafA (Fig. 4c).

**Figure 5:**
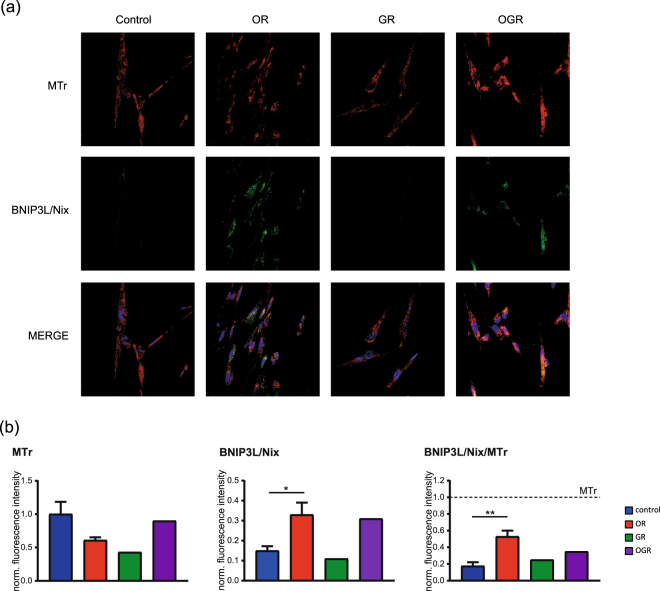
Mitochondrial content and degradation activity analyses (mitophagy) by confocal microscopy. (**a**) IMR90 cells stained with the mitochondrial marker MitoTracker red (MTr) and the mitophagy marker BNIP3L/Nix upon control, OR, GR and OGR. (**b**) Quantification of the MTr, BNIP3L/Nix as well as the BNIP3L/Nix to MTr ratio by measuring the total fluorescence. *p ≤ 0.05, **p ≤ 0.01. P-values were determined by one-way ANOVA with Dunnet’s multiple comparison’s test. Data represent mean ± s.e.m.

**Figure 6:**
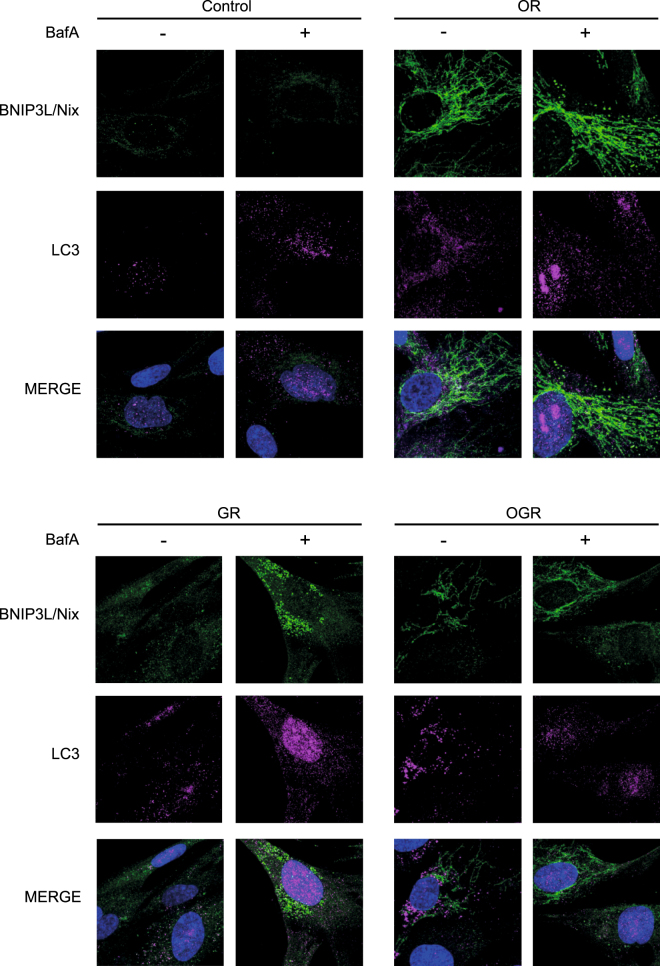
Mitophagy and autophagy analyses by confocal microscopy. (**a**) Primary human IMR90 cells cultivated under OR, GR and OGR for 24 h and 4 h prior to the harvesting cells were treated with and without BafA. Afterwards cells were fixed and immunostained with BNIP3L/Nix and LC3 antibodies and analyzed microscopically. Representative photographs of cells are shown.

We further investigated treatment-specific effects on mitophagy. Western Blotting analyses showed a significant decrease of BNIP3L/Nix protein levels at GR and increase at OR and OGR (Fig. 4b). Based on our findings, we further analyzed potential changes in the mitochondrial morphology, function and content. Therefore, we calculated the ratio between mitochondria marked for degradation and the total mitochondria by staining fibroblasts with MitoTracker red (MTr, mitochondrial marker) and BNIP3L/Nix (marked mitochondria for mitophagy). In line with our Western blotting results, OR significantly elevated the BNIP3L/Nix signal and the BNIP3L/Nix to MTr ratio. OGR showed similar effects, however not significantly (Fig. 5a and b). In addition, we analyzed the two degradative processes – autophagy and mitophagy – by microscopy. We employed the autophagy and mitophagy markers LC3 and BNIP3L/Nix, respectively, with and without BafA. Interestingly, BNIP3L/Nix is visible under all three conditions with the strongest effect observed upon OR. In line with our Western blotting autophagic activity analyses the microscopic results also show a treatment-specific induction of the autophagy and mitophagy upon OR and GR, respectively, but not under OGR (Fig. 6).

## Discussion

Metabolomics provides an analytical tool for the identification of pathway alterations and biosignature analyses. To our knowledge this is the first study that identifies pathways, metabolite level and ratio changes in human primary fibroblasts comparing OR, GR and OGR treatment. Our study indicates that the effects of OR, GR and OGR results in several significant metabolite level and ratio alterations, rearrangements of major energy metabolism pathways, regulators as well as an affected autophagic and mitophagic activity.

In the current study, we observed that GR, OR and OGR mainly affects major energy metabolism pathways. All treatments enrich for the ‘glycolysis’, ‘gluconeogenesis’ and the ‘pentose phosphate pathway’ (Fig. 1b) and alter many of its metabolite levels and ratios. We also measured a significant enrichment and upregulation of the ‘aspartate metabolism’ upon GR and OGR, which is involved in regulating ‘glycolysis’, ‘gluconeogenesis’, ‘citric acid cycle’ and ‘protein biosynthesis (amino acids)’^39,40^. However, the ‘citric acid cycle’ seems not to be systematically affected, rather specific metabolites are treatment-specifically changed. Interestingly, upon OR the only observed overrepresented and downregulated pathways were ‘glycolysis’ and the ‘pentose phosphate pathway’ (Fig. 1b). Furthermore, our analyses indicate that the metabolite alterations observed in OR differ from GR and OGR, as observed by the smaller amount of overlapping metabolites in OR with GR and OGR (Fig. S2). Taken together, these results provide evidence that fibroblasts grown upon OR, GR and OGR undergo a number of treatment-specific adaptations in key energy metabolic pathways.

The measured upregulation of the ‘aspartate metabolism’ under GR and OGR (Fig. 1b) may also affect linked pathways including the ‘purine and pyrimidine metabolism’. For instance, an altered ‘aspartate metabolism’ is thought to be involved in the pathogenesis of Alzheimer’s disease^41^. Aspartate is a substrate for a brain peptide – N-Acetylaspartylglutamic acid – meeting the criteria of a neurotransmitter. Its main mode of action is the activation of metabotropic glutamate receptor 3. However, the peptide’s activity on the N-methyl-D-aspartate (NMDA) receptor is controversially discussed^42,43^. The consequences of altered ‘aspartate metabolism’ for neurotransmission may require further investigations in neuronal cells.

Metabolite ratio changes can indicate protein level or enzymatic activity alterations. Interestingly, we could not confirm all our metabolite ratio alterations for the glycolytic enzymes by Western Blotting. Therefore, the ratio alterations might most likely be the result of enzymatic activity changes that we discussed below in more detail on GAPDH. However, our obtained results are in line with previous analysis showing an upregulation of many glycolytic metabolites and enzymes upon hypoxia in cancer cell lines^1,2^.

Our results demonstrate a significant increase in AMP levels under OGR. In addition, the ATP/AMP ratio is decreased and elevated upon OR and GR as well as OGR, respectively (Fig. 4a). An intracellular decline of the ATP/AMP ratio activates the AMPK, the major energy sensing protein. Therefore, the kinase is involved in regulating cellular energy metabolism pathways by sensing the nutrient and energy status. LKB1 is the major kinase phosphorylating the AMPK activation loop under energy stress conditions as demonstrated by biochemical and genetic analyses in worms, flies and mice^44^. Upon activation, AMPK downregulates anabolic pathways that would consume large amounts of energy through the inhibition of mTORC1^45,46^. In line with previous studies, our analyses revealed a significant activation of AMPK upon GR (Fig. 4b). Interestingly, there is still a high activation of AMPK upon GR indicating a pLKB1 independent activation of AMPK^7,47,48^. pAMPK also activates the glycolytic enzyme GAPDH, which in turn interacts with sirtuin 1 (SIRT1), stimulates its activation and thus, LC3 deacetylation and induction of autophagy^19^. Autophagy is a catabolic process consisting of multiple sequential steps, by which proteins and organelles can be degraded. Although the major function of autophagy is the elimination of misfolded, highly oxidized and damaged proteins and organelles, it is also involved balancing cellular energy. We further observed a metabolite ratio alteration of glyceric acid 1,3-biphosphate/glyceraldehyde 3-phosphate upon GR pointing towards an enzymatic activity change of GAPDH. GAPDH is activated by a decreased NADH/NAD ratio – which we indeed confirmed with our analyses – and activates SIRT1 in a pAMPK-dependent manner^19^. In addition, we observed significant alterations of the autophagic degradation activity (autophagic flux) based on LC3-II protein levels (Fig. 4c). Our results indicate an increased autophagosome number even without BafA in GR and OGR without a change in the autophagic degradation activity or autophagic flux under the latter one. Taken together, GR induced the autophagosome formation indicated by an increased pAMPK/AMPK ratio and elevated LC3-II protein levels upon BafA treatment.

In conclusion, we suggest that our metabolomics approach coupled with further biochemical and microscopic analyses revealed that energy producing catabolic pathways are induced upon GR, whereas energy-consuming anabolic processes are largely downregulated upon OR (Fig. 1 and Fig. S6). In detail, OR increases the ATP/AMP ratio and thus inhibits the AMPK, whereas GR reduces the ATP/AMP ratio leading to an AMPK activation – the latter also inhibiting mTOR. Therefore, the AMPK and mTOR activity promotes catabolism and anabolism, respectively. Autophagy and glycolysis are major catabolic pathways. Therefore, the induction of autophagy upon GR as well as the opposite regulation of glycolytic metabolites upon OR and GR further supports our conclusion.

An integral part of the metabolic adaptation towards OR due to a hypoxic microenvironment in cancer cells is the HIF1α mediated gene expression involved in shifting the metabolism towards anaerobic glycolysis^20^. HIF1α increases anaerobic metabolic flux and survival by expressing Glut1 and glycolytic enzymes^21^. In line with these results, we also mainly observed that limited oxygen supply stabilizes HIF1α and then binds to HIF1β to form an active HIF1 complex inducing its target genes; thus leading to complex metabolic rearrangements^25,26,49^. Moreover, HIF also regulates mitochondrial functions. HIF1 stabilization through hypoxia leads to elevated PDH kinase 1 protein levels, which in turn inactivates PDH; thus limiting the conversion of pyruvic acid to acetyl-CoA in the mitochondria. Consequently, PDH kinase 1 induction decreases the ‘citric acid cycle’ activity and reduces oxygen consumption^25,27^. In addition and in line with our results, HIF1α rather induces mitophagy by increased protein expression of BNIP3L/Nix than autophagy^22,24^. Similar to cancer cells, in the present study primary human fibroblasts under OR and OGR demonstrate a strong elevation of HIF1α as well as Glut1 and BNIP3L/Nix protein levels. On the other hand, GR significantly decreased BNIP3L/Nix protein levels (Fig. 4b). Moreover, we observed an increased BNIP3L/Nix to MTr ratio upon OR indicating an elevated mitochondrial degradation (Figs 4, 5 and 6). Taken together, our present results indicate an accelerated mitochondrial degradation or mitophagy upon OR and OGR and autophagy in GR.

In conclusion, we submit that the observed treatment-specific cellular alterations upon OGR are due to a downregulation of both anabolic and catabolic processes. Additionally, the observed high mitophagic activity is most likely occurring independently of LC3-II. Another important observation was a concomitant treatment-specific induction of HIF1α, Glut1 and BNIP3L/Nix proteins.

Taken together, our present study indicates a high degree of metabolite and protein changes adjusting towards a cellular energy demand upon OR, GR and OGR. This study proposes that the interplay of autophagy/mitophagy and changes in the metabolome are major contributing sources for the cellular adaptation to nutrient and oxygen shortage. Furthermore, the present study suggests an induction and suppression of catabolic and anabolic processes, respectively, protecting and providing cell survival upon oxygen and glucose reduction.

Furthermore, we suggest that our results have a strong impact for several disorders with well-known bioenergetics perturbations including ageing and age-associated neurodegeneration such as Alzheimer’s disease and Parkinson’s disease. Our results gain further insights into the basic cellular mechanism and adaptive regulations that help the cells to survive.

For instance, cancer cells often switch from OXPHOS to aerobic glycolysis in order to generate ATP, which is described as the Warburg effect^14^. However, this switch seems not to be a result of a defective mitochondrial respiration, it rather occurs through various factors including a hypoxic microenvironment thereby inducing HIF1α signaling^20,50,51^. Nutrient shortage due to cancer malignancy and its high proliferation rate is a subsequent result. A hallmark of Parkinson’s disease is the loss of dopaminergic neurons of the midbrain substantia nigra. A combination of mitochondrial dysfunction and oxidative stress is widely believed to underlie the pathogenesis of sporadic Parkinson’s disease^49,52–54^; thus, leading to bioenergetics imbalances. In addition, normal ageing as well as dementia are causing bioenergetics perturbations, too, resulting in a need of cellular adaptive regulations.

Not only in cancer but also in dementia, in ageing, in heart failure, and in Parkinson’s disease, cells might profit from and adapt towards the bioenergetics perturbations by the Warburg effect. In addition, glycolysis and the truncated citric acid cycle provide not only ATP, but also metabolic intermediates. Therefore, the required macromolecules like nucleic acids, lipids and proteins can be metabolized for cellular growth and proliferation. For instance, several amino acid precursors are derived from transamination of mitochondrial metabolic intermediates. Oxalacetic acid can be transaminated to produce asparatic acid and oxoglutaric acid can be transformed into glutamate, which can further be transferred to proline, arginine and glutamine^52^. In addition, metabolic intermediates can also fuel the citric acid cycle, which could be the case upon GR. Further experiments including mitochondrial respiration measurements are required to confirm our findings and to test our hypothesis.

We further submit that two adaptation mechanisms are involved in coping with glucose and oxygen stress: Alterations in major energy metabolism pathways accompanied by increased autophagy in GR as well as mitophagy in OR and OGR. Our model indicates that upon OR HIF1α leads to glycolytic enzyme, Glut1 and BNIP3L/Nix protein expression. Moreover, these alterations rather would lead to an increased aerobic glycolysis than mitochondrial respiration and further to increased mitophagy. Taken together, cells would adapt to OR by producing most of the metabolic intermediates through the aerobic glycolysis and mitophagy. The energy will be generated through aerobic glycolysis and the intermediates can further be used in order to synthesize new macromolecules; thus supporting cellular survival (Fig. S5).

In contrast, we submit another mechanism for GR. pAMPK as well as a decrease in NAD/NADH metabolite ratio activates GAPDH, which in turn induces SIRT1 and autophagy^19^. The metabolic intermediates gained by this mechanism can further fuel the citric acid cycle; thus leading to normal mitochondrial respiration and energy production. Furthermore, new macromolecules that support cellular survival can be synthesized by metabolic intermediates obtained by autophagy (Fig. S5).

Oxygen and glucose reduction is a major correlate of a diminished blood supply (hypoperfusion) and might have a strong impact on major energy metabolism pathways. In addition, cells deprived in energy sources undertake large scale rearrangements in key metabolic pathways to counter balance nutrient deficiency^2^. However, the basic biomolecular pathways and consequences of these adaptations in primary cells remain largely unknown. We therefore submit that our cellular model meets the criteria for a basic *in vitro* model of hypoperfusion.

We only assessed our metabolomics profiling, biochemical and microscopic analyses in human fibroblasts. We preferentially used these cells in our study in order to investigate the basic cellular adaptation mechanisms upon GR, OR and OGR. Future metabolomics analyses of other relevant cell models – including relevant neuronal and cardiac cells – will further our understanding of specific adaptive mechanisms upon energy imbalances.

## Materials and Methods

All cell culture media and supplements reagents including high-glucose Dulbecco’s Modified Eagle’s Medium (DMEM), Dulbecco’s Phosphate-Buffered Saline (PBS), fetal calf serum (FCS) were from Invitrogen unless otherwise stated. Laboratory chemicals and biochemicals were purchased from Sigma at the highest available purity. Bafilomycin A (BafA) was from Biozol.

### Cell culture

Primary human fibroblasts IMR90 were purchased from Coriell Institute for Medical Research and cultivated as previously described^55^. Briefly, IMR90 cells were maintained in phenol red (pH indicator) containing Dulbecco’s modified Eagle’s medium (DMEM) with high glucose (4.5 g/L) and 1 mM sodium pyruvate and supplemented with 10% heat-inactivated fetal calf serum (FCS), antibiotics and antimycotics. For routine culture, cells were grown on 10 cm dishes and maintained at 37 °C in a humidified atmosphere containing 5% CO_2_, passaged twice a week. Experiments were carried out after cells reached the population doubling level (PDL) 20–25, which was determined exactly as previously described^53,55,56^.

For all assays cells were plated at a density of 0.5 × 10^6^ cells per 10 cm dish and were grown in full medium for 24 hours. After that the medium and cultivation conditions were changed to generate four experimental paradigms for 24 h: control, 21% oxygen and 4.5 g/L glucose); oxygen reduction (OR), 1% oxygen and 4.5 g/L glucose; glucose reduction (GR), 20% oxygen and 0 g/L glucose; oxygen-glucose-reduction (OGR), 1% oxygen and 0 g/L glucose.

All treatment groups were visually inspected using light microscopy by two independent researchers, both of them reporting no visible signs of morphological changes. Additionally, cleaved Caspase 3 (cleaved Casp 3), a well-established marker of apoptosis, was determined by immunoblotting. Salermide (a SIRT1 and SIRT2 inhibitor) was used as a positive control.

### Immunoblotting

24 hours post-treatment cells were washed with ice cold PBS and harvested with lysis buffer (0.6 mM Tris-HCl, pH 6.8; 2% sodium dodecyl sulfate and 10% sucrose plus protease- and phosphatase inhibitor) and briefly sonicated. Protein concentration was determined using BCA kit (Pierce) according to manufacturer’s protocol. 10 µg of total cell lysate were loaded on 12–15% SDS-PAGE and separated with Mini protean III system (Bio-Rad). Afterwards, proteins were transferred onto nitrocellulose membrane by electroblotting. Following 30 min incubation with 5% low fat milk in TBST membranes were incubated with the following primary antibodies: rabbit anti-HIF1α (1:1000, Novus Biochemicals); rabbit anti-BNIP3/Nix (1:1000, Cell signaling); rabbit anti-LC3b (1:1000, Sigma-Aldrich); rabbit anti-phospho-LKB (1:1000, Cell signaling); rabbit anti-LKB (1:1000, Cell signaling*)*, rabbit anti-phospho-AMPK (1:1000, Cell signaling); rabbit anti-AMPK ((1:1000, Cell signaling); mouse anti-phospho- p70S6K (1:1000, Cell signaling); rabbit anti- p70S6K (1:1000, Cell signaling); rabbit anti-Glut1 (1:1000, Cell signaling); rabbit anti-caspase 3 (1:1000, Cell signaling); glycolysis antibody sampler Kit: rabbit anti-HKII, rabbit anti-PFKP, rabbit anti-GAPDH, rabbit anti-PKM2, rabbit anti-LDHA, rabbit anti-PDH (1:1000, Cell signaling); Anti-α-tubulin (1:1000; Sigma-Aldrich) immunoreactive signal was used as a control for equal protein loading. Primary antibodies were detected with horseradish peroxidase conjugated secondary antibodies. Immunoreactive bands were developed using commercial kits (Enhanced Chemiluminescence Plus from Amersham Pharmacia Biotech, Piscataway, NJ, USA) and scanned (Fuji Las-3000 Dark Box, Fuji, Tokyo, Japan). The densitometric analysis of the immunoreactive bands were performed using Aida Image analysis Software (Raytest, Straubenhardt, Germany).

### Immunocytochemistry

IMR90 cells were grown on glass cover slips and fixed with 4% paraformaldehyde. Unspecific epitopes were blocked with 3% BSA and cells were subsequently permeabilized with 0.1% Triton X-100. At this point, cells were incubated overnight with primary antibodies (rabbit anti-LC-3, diluted 1:100 with PBS, rabbit anti-BNIP3/Nix, diluted 1:250 with PBS) in PBS containing 1% BSA. After that, cells were incubated with secondary goat anti-rabbit Cy3- and Cy5-coupled antibody for 2 hours at room temperature. Cell nuclei were counterstained with 1 µg/mL 4, 6-diamidino-2-phenylindole (DAPI, Sigma). Cultures were analyzed and photographed using a confocal laser-scanning microscope LSM710 (Zeiss).

### Assessment of mitochondrial morphology

Mitochondrial morphology in intact IMR90 cells was determined using mitochondrial-specific dye MitoTracker Red as previously described^54^. Briefly, IMR90 cultivated on coverslips were challenged with OR, GR and OGR for 24 hours and then incubated with 100 nM MitoTracker Red for 4 hours before they were fixed with −80 °C methanol for 20 min, rinsed with PBS and mounted on a microscope slide. The stained cells were serially photographed with a confocal laser-scanning microscope and identical acquisition parameters in all cases. The obtained photographs were analyzed using Image J analysis software (Universal Imaging, Perkin Elmer).

### Metabolomics data acquisition and statistical analysis

#### Sample preparation

IMR90 cultivated on 10 cm dishes were challenged with OR, GR and OGR for 24 h and then rinsed twice with PBS on ice. Soluble metabolites were isolated using the previously published protocol^17,57^.

### Targeted metabolomics analysis

Samples were resuspended using 20 μl liquid chromatography-mass spectrometry grade water. Ten microliters were injected and analyzed using a 5500 QTRAP triple quadrupole mass spectrometer (AB/SCIEX, Framingham, MA, USA) coupled to a Prominence UFLC high-performance liquid chromatography system (Shimadzu, Columbia, MD, USA) via selected reaction monitoring of a total of 254 endogenous water-soluble metabolites for steady-state analyses of samples. Samples were delivered to the mass spectrometer via normal phase chromatography using a 4.6-mm i.d × 10 cm Amide Xbridge HILIC column (Waters, Milford, MA, USA) at 350 μl min^−1^. Gradients were run starting from 85% buffer B (high-performance liquid chromatography grade acetonitrile) to 42% B from 0 to 5 min; 42% B to 0% B from 5 to 16 min; 0% B was held from 16 to 24 min; 0% B to 85% B from 24 to 25 min; 85% B was held for 7 min to re-equilibrate the column. Buffer comprised 20 mM ammonium hydroxide/20 mM ammonium acetate (pH = 9.0) in 95:5 water: acetonitrile. Some metabolites were targeted in both positive and negative ion modes for a total of 285 selected reaction monitoring transitions using positive/negative polarity switching. Electrospray ionization voltage was +4900 V in positive ion mode and −4500 V in negative ion mode. The dwell time was 4 ms per selected reaction monitoring transition and the total cycle time was 1.89 s. Approximately 9–12 data points were acquired per detected metabolite. Peak areas from the total ion current for each metabolite-selected reaction monitoring transition were integrated using the MultiQuant v2.0 software (AB/SCIEX).

### Identification of significant metabolite level alterations

Metabolite intensities were sample normalized and z-scored for further statistical analysis. Significant metabolite level changes upon OR, GR and OGR were identified by PLS-DA and SAM using MetaboAnalyst as previously published^58^. The quality of the PLS-DA models was assessed for R^2^, Q^2^ and accuracy values with VIP-score ≥ 1.0, for SAM with q ≤ 0.05 and false discovery rate (FDR) ≤ 0.05 (adjusted p-value). PLS-DA is prone to overfitting and therefore the quality of the model is assured by cross-validation. Therefore, especially the R^2^ and Q^2^ are calculated as they describe the goodness of fit or explained variation and the predicted variation or the quality of prediction, respectively. The values indicate how well the PLS-DA model is able to mathematically reproduce the data in the dataset. In general, a poorly and well fit model is described by R^2^ ~0.20–0.30 and ~0.80–0.90, respectively. Furthermore, a good PLS-DA model is described by Q^2^ ≥ 0.50 and an outstanding PLS-DA model is defined by ≥0.90. The PLS-DA VIP-score ranks metabolites according to their importance in separating two classes. Therefore, for the identification of metabolite changes characteristic for group separation, metabolites with VIP-scores ≥ 1.0 are considered important for group separation and were selected for subsequent data analysis. To further increase the robustness of the analyses, we combined the PLS-DA with results obtained from a SAM analysis in order to determine a reliable list of metabolites that significantly contribute to the respective condition. We improved robustness of our data analyses and increased confidence in significantly altered metabolites as well as in the subsequent analyses of overrepresented metabolite set enrichment analysis (MSEA) by only considering the overlap between the two different statistical methods. The obtained list of significantly altered metabolites was also separated into up- and downregulated levels which were subsequently used for further metabolomics analyses.

### Identification of significantly overrepresented pathways

Metabolomics pathway analyses were performed using MetaboAnalyst applying metabolite set enrichment analysis (MSEA) for overrepresentation analyses. Pathways were considered affected if they were significantly enriched (FDR ≤ 0.05). Therefore, the up- and downregulated metabolites obtained by SAM and PLS-DA were (see also Table S2–4) separately used for MSEA, thus resulting in up- and downregulated overrepresented pathways.

### Calculation of metabolite pair ratios

Selected pairs of metabolites (metabolite ratios) were calculated as described previously^17^.

### Data availability

The datasets generated during and/or analyzed during the current study are available from the corresponding author on reasonable request.

## Electronic supplementary material

Supplementary Information

Table S1

Table S2

Table S3

Table S4
