# Author Correction: Metabolomics profiling reveals differential adaptation of major energy metabolism pathways associated with autophagy upon oxygen and glucose reduction

**DOI:** 10.1038/s41598-018-28914-9

**Published:** 2018-08-10

**Authors:** Katja Weckmann, Philip Diefenthäler, Marius W. Baeken, Kamran Yusifli, Christoph W. Turck, John M. Asara, Christian Behl, Parvana Hajieva

**Affiliations:** 1 Institute of Pathobiochemistry, Johannes Gutenberg University, Medical School, Duesbergweg 6, 55099 Mainz, Germany; 2 Department of Translational Research in Psychiatry, Max Planck Institute of Psychiatry, Kraepelinstr. 2–10, 80804 Munich, Germany; 3 Division of Signal Transduction/Mass Spectrometry Core, Beth Israel Deaconess Medical Center, Boston, Massachusetts, USA and Department of Medicine, Harvard Medical School, Boston, Massachusetts, USA

Correction to: *Scientific Reports* 10.1038/s41598-018-19421-y, published online 05 February 2018

In Figure 4c, the heading “Autophagic ac”vity analysis” should read “Autophagic activity analysis”.

Additionally, the “+” and “−” symbols are reversed in the displayed Western blots and graphs.

The correct Figure 4 appears below as Figure 1.

**Figure 1:**
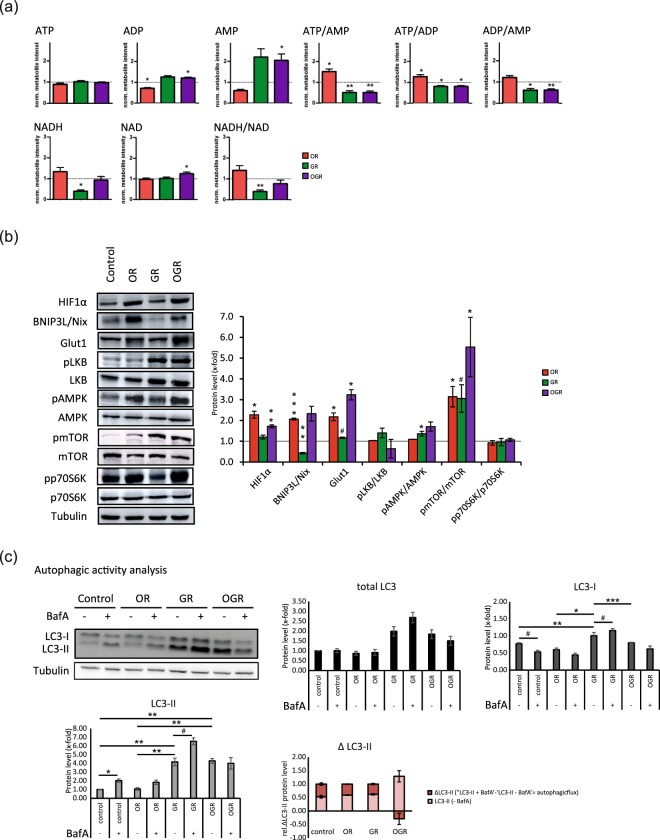
(**a**) Energy-status analyses of ATP, ADP, AMP, NAD and NADH with the related metabolite ratios ATP/AMP, ATP/ADP, ADP/AMP, and NADH/NAD. *p ≤ 0.05; **p ≤ 0.01. P-values were determined by Student’s t-test. Error bars represent s.e.m. N = 5 per group. (**b**) Western Blotting analyses of markers for GR and OR and proteins involved in the cellular energy metabolism. IMR90 cells were subjected to OR, GR and OGR for 24 h. After that cells were harvested and total cell lysate was analyzed using Western blotting and immunodetected with indicated antibodies. Tubulin was used as a loading control. For the densitometric quantification of the immunoreactive bands the absolute values measured were first normalized to tubulin and the resulting values to the control, which was set as 1. ^#^≤ 0.10, *p ≤ 0.05; **p ≤ 0.01, ***p ≤ 0.001. P-values were determined by one-way analysis of variance (ANOVA) with post-hoc Tukey honestly significant difference (HSD) test. Error bars represent s.e.m. N = 3 per group. (**c**) Autophagic degradation activity analyses upon OR, GR and OGR compared to control measured by Western blotting analyzing LC3 and LC3-II protein levels and LC3-II protein turnover with and without BafA. The autophagic degradation activity (autophagic flux) was determined by the following calculation: ΔLC3-II = ‘LC3-II + BafA’ - ‘LC3-II - BafA’. Tubulin was used as a loading control. ^#^≤ 0.10, *p ≤ 0.05, **p ≤ 0.01. P-values were determined by one-way ANOVA with post-hoc Tukey HSD test. Error bars represent s.e.m. N = 3 per group.

