# Supplementary material for: Metabolomics profiling reveals differential adaptation of major energy metabolism pathways associated with autophagy upon oxygen and glucose reduction
Source: Sci Rep. 2018 Feb 5;8:2337. doi: 10.1038/s41598-018-19421-y (PMC5799166; doi:10.1038/s41598-018-19421-y)
Supplement: Supplementary file 1 — Supplementary Information [file 41598_2018_19421_MOESM1_ESM.pdf]

Metabolomics profiling reveals differential adaptation of major energy metabolism pathways associated with autophagy upon oxygen and glucose reduction

Katja Weckmann[1], Philip Diefenthäler[1], Marius W. Baeken[1], Kamran Yusifli[1], Christoph W. Turck[2], John M Asara[3], Christian Behl[1] and Parvana Hajieva[1]*

Supplementary information

Figure S1: Western Blotting analyses of the apoptotic marker Caspase 3

Figure S2: Analyses of overlapping metabolites

Figure S3: Metabolite ratio analyses of glycolysis metabolites

Figure S4: Metabolite ratio analyses of citric acid cycle metabolites

Figure S5: The suggested mechanism of adaptation

Figure S6: Metabolite set enrichment analysis (MSEA) and pathway regulation

Figure S7: Full length Western Blot images of figure 2b

Figure S8: Full length Western Blot images of figure 4b

Figure S9: Full length Western Blot images of figure 4c

Figure S10: Full length Western Blot images of figure S6

Supplementary figures

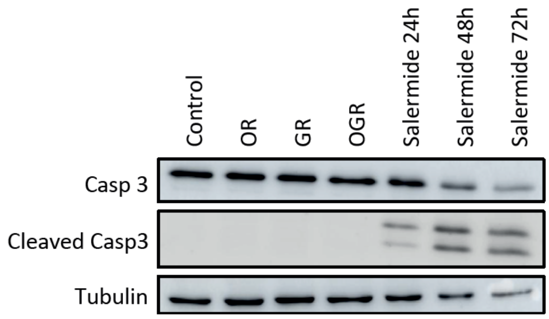

Figure S1. Western Blotting analyses of the apoptotic marker Caspase 3 (Casp 3), which in its cleaved form is an executer of apoptosis. Salermide (a SIRT1 and SIRT2 inhibitor) was used as a positive control and fibroblasts were treated for 24h, 48 and 72h.

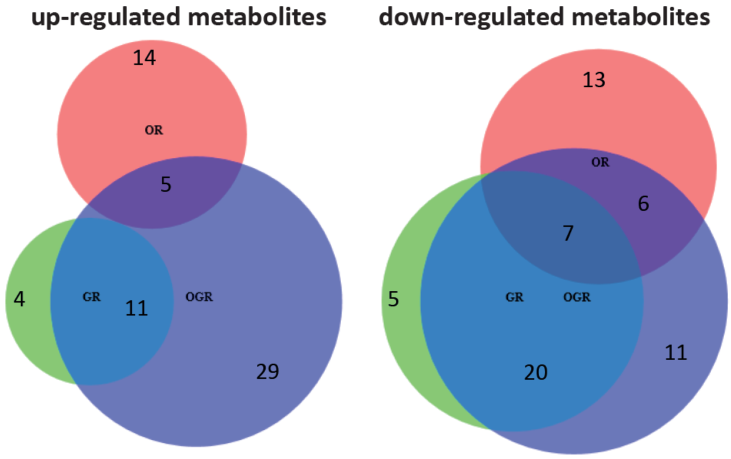

Figure S2. Analyses of overlapping metabolites upon OR, GR and OGR. Significantly up- and downregulated metabolites of each treatment were compared by a VENN-diagram.

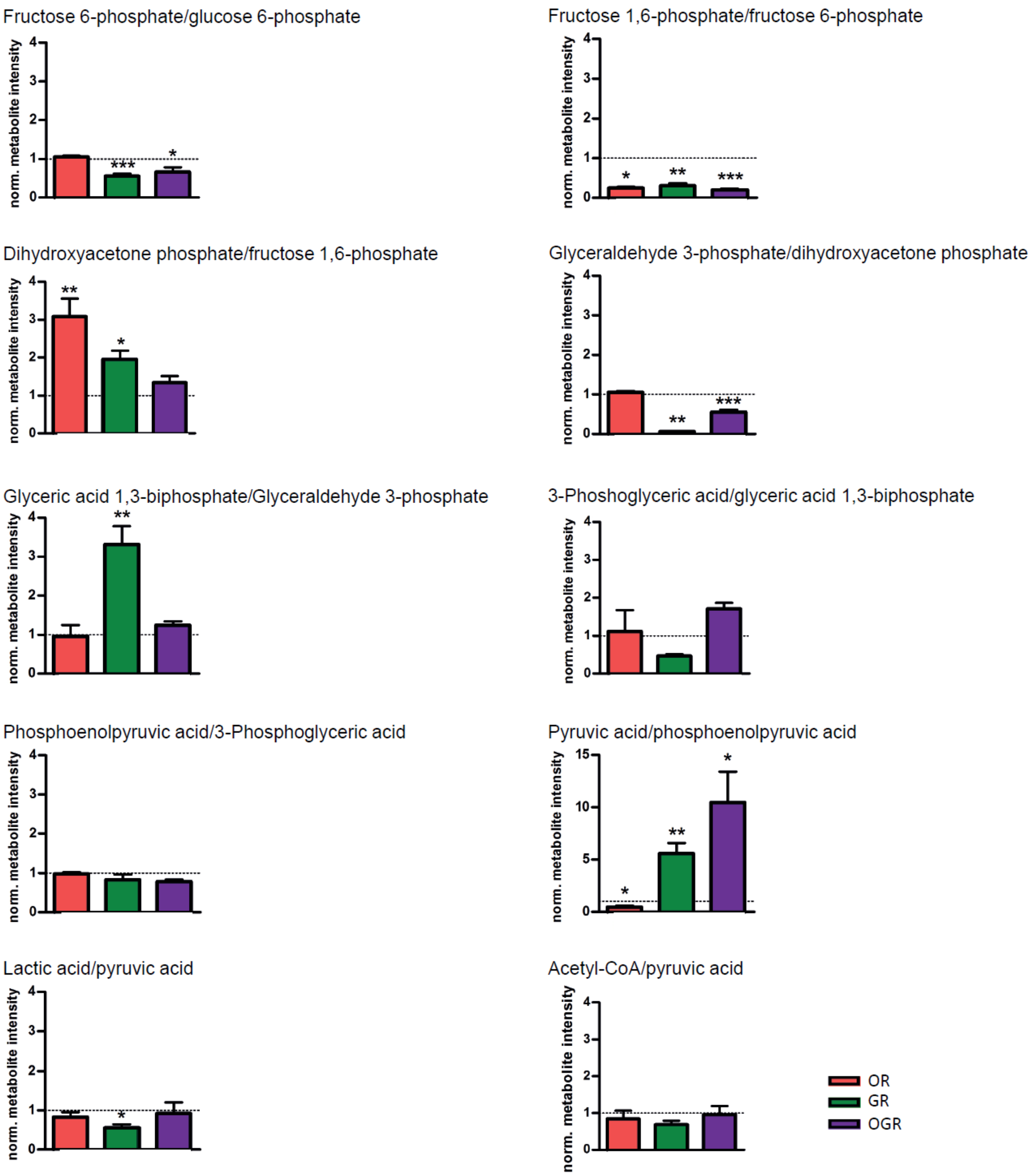

Figure S3. Metabolite ratio analyses of glycolysis metabolites. Ratio levels for selected pairs of metabolites (metabolite ratios) were calculated indicating alterations of enzyme activity or protein expression. N=5 per group. *, p≤0.05; **, p≤0.01; ***, p≤0.001. P-values were determined by Student's t-test. Error bars represent s.e.m. N=5 per group.

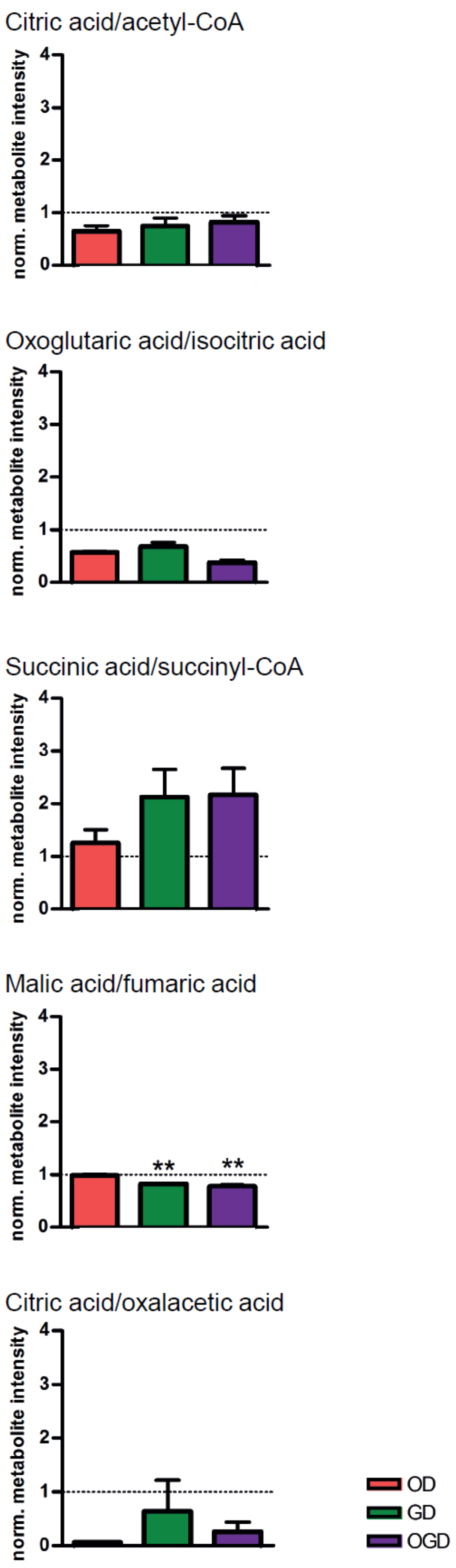

Figure S4. Metabolite ratio analyses of citric acid cycle metabolites. Ratio levels for selected pairs of metabolites (metabolite ratios) were calculated indicating alterations of enzyme activity or protein expression. N=5 per group. *, p≤0.05; **, p≤0.01; ***, p≤0.001. P-values were determined by Student's t-test. Error bars represent s.e.m. N=5 per group.

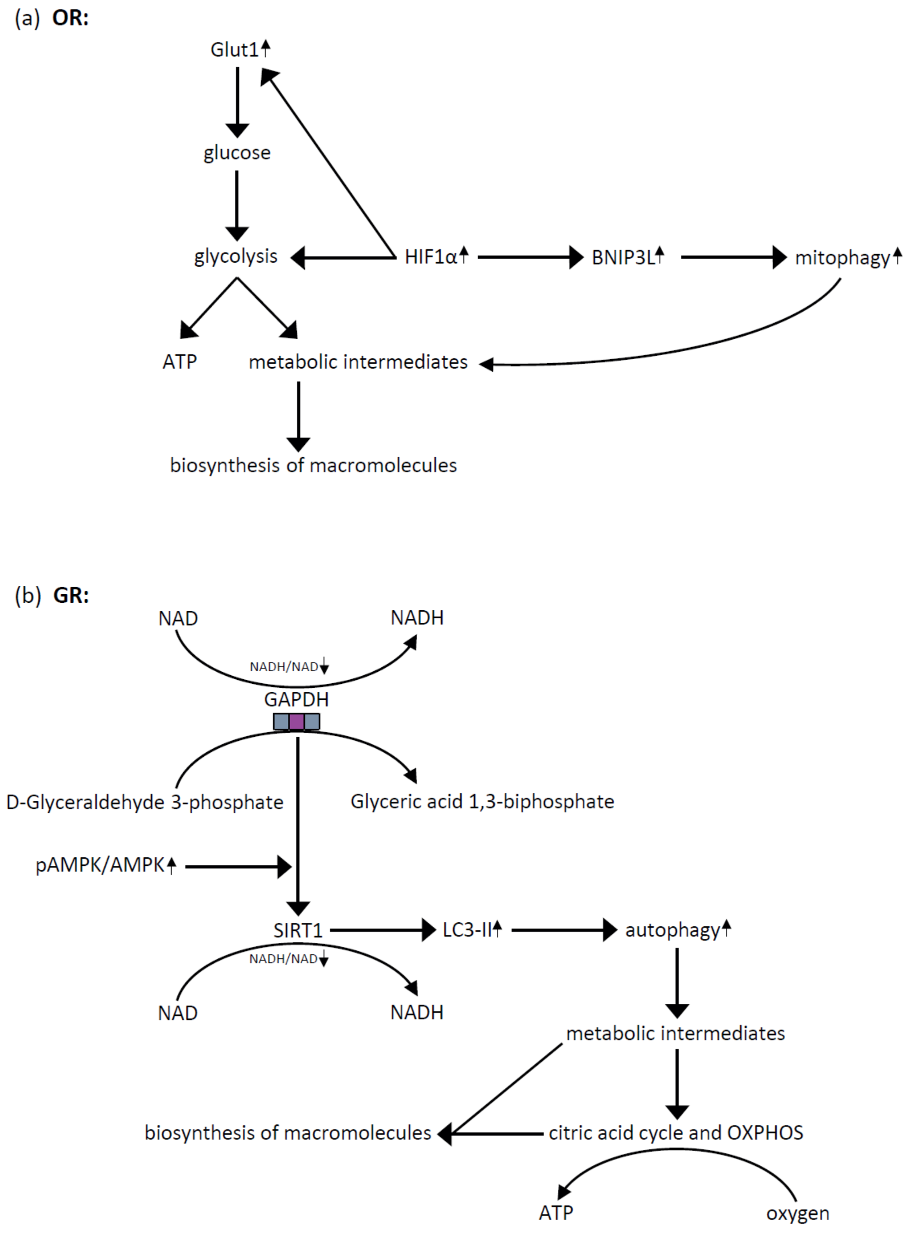

Figure S5. Model indicating that (a) upon OR HIF1α leads to glycolytic enzyme, Glut1 and BNIP3L/Nix protein expression. Alterations would rather lead to an increased aerobic glycolysis than mitochondrial respiration and further to increased mitophagy. Cells would adapt to OR by producing most of the metabolic intermediates through the aerobic glycolysis and mitophagy. The energy will be generated through aerobic glycolysis and the intermediates can further be used in order to synthesize new macromolecules; thus supporting cellular survival. B) Upon GR pAMPK as well as a decrease in NAD/NADH metabolite ratio activates GAPDH which in turn induces SIRT1 and therefore autophagy. The metabolic intermediates gained by this mechanism can further fuel the citric acid cycle; thus leading to normal mitochondrial respiration and energy production. Metabolic intermediates obtained by autophagy can further be used in order to synthesize new macromolecules that support cellular survival.

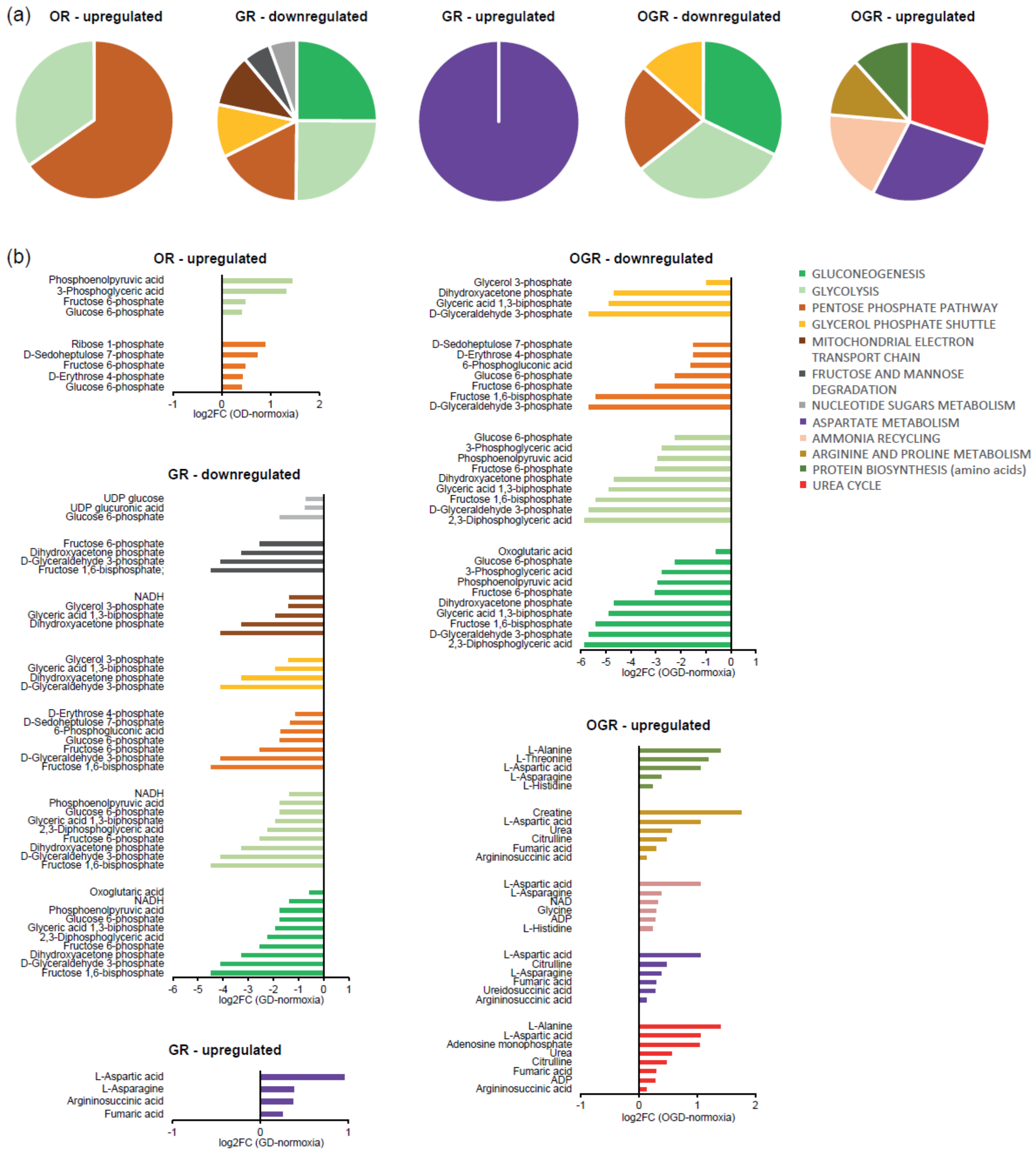

Figure S6. A) Metabolite set enrichment analysis (MSEA) and pathway regulation upon OR, GR and OGR. Significantly up- and downregulated metabolites from Table S2-S4 were separately analyzed for MSEA (OR – upregulated metabolites, GR – downregulated metabolites, GR – upregulated metabolites, OGR – downregulated metabolites, OGR – upregulated metabolites).

B) Fold change (FC) analysis of each up- and downregulated pathway upon OR, GR and OGR showing the log2-transformed metabolite FC's (OR-control; GR-control and OGR-control). N=5 per group.

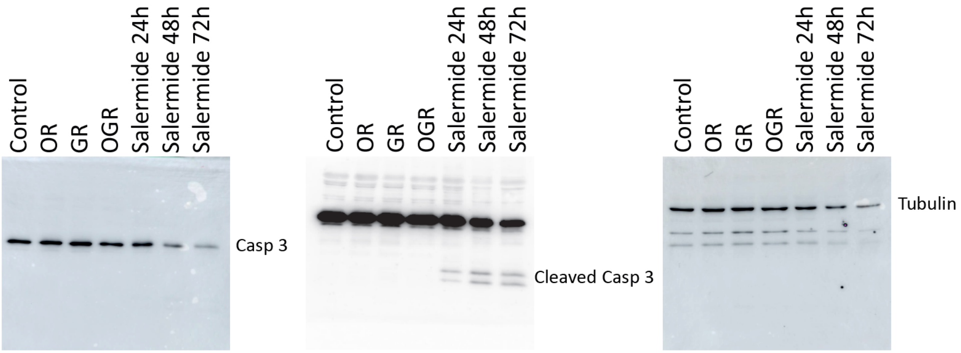

Figure S7. Full length Western Blot images of figure 2b.

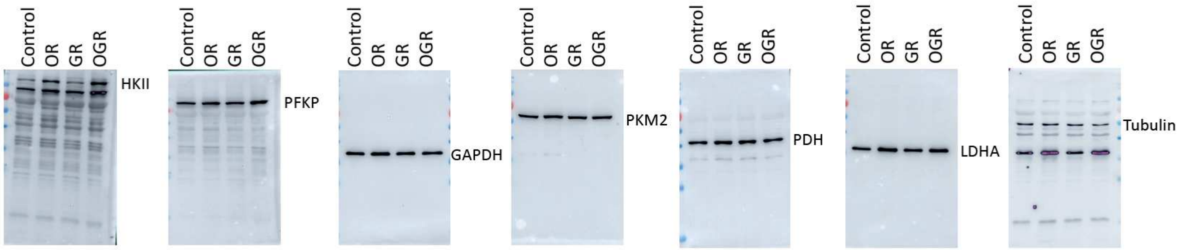

Figure S8. Full length Western Blot images of figure 4b.

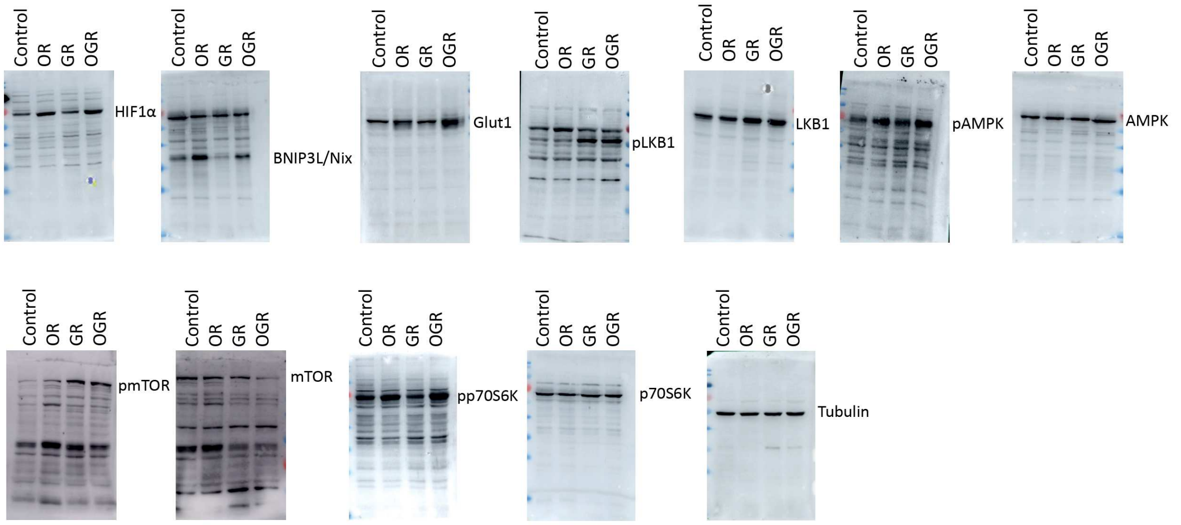

Figure S9. Full length Western Blot images of figure 4c.

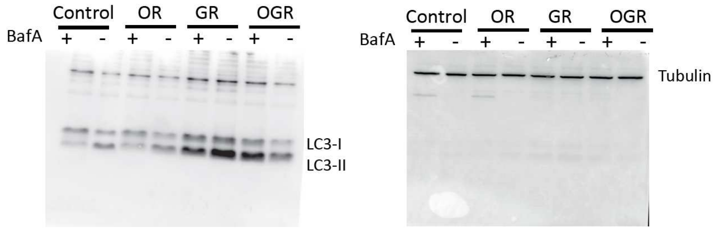

Figure S10. Full length Western Blot images of figure S6.
